# Flu Vaccine Uptake in Caregivers and Noncaregivers: Implications for Policy and Practice

**DOI:** 10.5888/pcd20.220125

**Published:** 2023-01-05

**Authors:** Nicholas R. Mercado, Kenneth Knapp, Erin D. Bouldin, Elizabeth D. Drugge

**Affiliations:** 1 Department of Health Humanities and Bioethics, University of Rochester School of Medicine and Dentistry, Rochester, New York; 2 Department of Public Health, New York Medical College, Valhalla, New York; 3 Department of Internal Medicine, University of Utah, Salt Lake City, Utah

## Abstract

**Introduction:**

Caregivers are a critical and highly used health care resource. Caregivers may experience adverse health outcomes and practice less self-care, including obtaining vaccinations, while serving in their roles. Influenza (flu) is a common infectious disease responsible for millions of doctor visits, hospitalizations, and approximately 43,000 US deaths annually that can largely be prevented by receiving seasonal vaccinations. We aimed to estimate and compare the prevalence of flu vaccination among caregivers and noncaregivers. We hypothesized that caregivers would have a lower prevalence of flu vaccination than noncaregivers and that sociodemographic variables, health-related variables, and caregiving-specific characteristics would be associated with vaccine uptake.

**Methods:**

We analyzed Behavioral Risk Factor Surveillance System data from 2016 through 2018 on 154,170 respondents from 27 US states and the District of Columbia. We used bivariate analysis to estimate the difference in flu vaccination uptake among caregivers and noncaregivers and logistic regression to estimate differences after adjusting for individuals’ characteristics.

**Results:**

Logistic regression indicated no significant difference in flu vaccine uptake between caregivers and noncaregivers. Caregiving characteristics such as years in a caregiver role, weekly time spent caregiving, relationship to care recipient, and recipient’s risk for flu complications were also nonsignificant. Sociodemographic factors such as marital status, income, health insurance coverage, and race had a significant impact on flu vaccine uptake.

**Conclusion:**

Although no significant differences in flu vaccine uptake were found between caregivers and noncaregivers, flu vaccine coverage remains low in both groups. Evidence-based programs and policies to improve vaccine coverage in the caregiver and general populations remains a public health priority.

**Summary**

**What is already known on this topic?**

Influenza (flu) is a highly contagious infectious disease that can result in serious illness and death. Family caregivers often experience adverse health outcomes and practice less self-care, which may include forgoing annual flu vaccines.

**What is added by this report?**

No significant difference in flu vaccine uptake between caregivers and noncaregivers was found; however, overall flu vaccine uptake rate remains low.

**What are the implications for public health practice?**

Given the important role caregivers serve in the health care system, efforts must be made to improve flu vaccine uptake to protect their health and the health of their care recipients.

## Introduction

Family caregivers (hereinafter, “caregivers”) are a critical and highly used national health care resource in the United States (1). In addition to providing general support, caregivers provide aid or assistance to family members or friends with health conditions or disabilities (2); help with everyday activities of daily living such as toileting, bathing, and feeding (3); provide routine nursing-type care like lifting and moving (4); and help with medication management (5). In 2020, an estimated 53 million US adults reported providing care to a friend or family member, which was approximately a 22% increase between 2015 and 2020 (1).

Caregiving can bring benefits to the caregiver by providing a sense of purpose or meaning (1). However, caregiving may also result in caregiver burden. Caregiver burden is any negative impact or suffering perceived by the caregiver and may occur in the physical, emotional, social, financial, or spiritual domains (6). As a result of caregiver burden, caregivers have a higher prevalence of poor self-care behaviors and ignoring self-health (7), including forgoing preventive health care such as visiting a primary care physician for annual well visits, getting enough rest, or taking care of oneself when sick (8). Thus, it is possible that caregivers also may less frequently receive recommended vaccines.

Influenza, or “flu,” is an acute respiratory disease caused by a virus that can lead to severe illness, hospitalization, or even death (9). People who are at high risk for developing severe influenza leading to pneumonia include young children (10), immunocompromised adults (11), adults with chronic obstructive pulmonary disease (12), and adults aged 50 years or older (13). The most effective method for preventing the spread of influenza and the development of influenza disease is the annual influenza vaccine (14). Annual flu vaccines, along with other primary prevention methods, can help reduce the incidence of the flu, especially in high-risk patients and their caregivers (15).

Despite several contraindications for receiving the live attenuated flu vaccine, the inactivated flu vaccine is recommended for nearly all people aged 6 months or older (14). Flu vaccine uptake in the US is higher among older than younger adults, partly because of greater perceived susceptibility to severe flu complications (16). Disparities exist in vaccine uptake between Black or African American people and people of other races, which, in part, is attributed to mistrust of the health care system (17). Finally, health status is related to vaccination; people who have good or better self-reported health forgo annual flu vaccines more often than individuals who have poor self-rated health (18).

Caregivers are advised to be up to date on their vaccinations whether they care for children or adults (19,20). However, the focus of investigating flu vaccine uptake has been on professional caregivers, such as health care workers (21), or strictly on patients (22); there has yet to be an attempt to investigate the vaccination habits of family caregivers. The purpose of this study was to investigate the uptake of the influenza vaccine among caregivers and evaluate whether the influenza vaccination rates differed from noncaregivers. Our aims were to estimate the prevalence of flu vaccination among caregivers and noncaregivers and to identify factors associated with vaccination. We hypothesized that caregivers would have a lower prevalence of vaccination than noncaregivers and that both sociodemographic variables (race and ethnicity, sex, educational attainment, age, income) and caregiving-specific characteristics would affect influenza vaccine uptake (17,23).

## Methods

### Data Source

This study used 3 years of data (2016–2018) from the Behavioral Risk Factor Surveillance System (BRFSS). The BRFSS is the US state-based system of telephone surveys that collects data about noninstitutionalized residents aged 18 years or older regarding their health-related risk behaviors, chronic health conditions, and use of preventive services (24). The BRFSS questionnaire has 3 parts: 1) the core instrument, 2) the optional modules, and 3) the state-added questions. All jurisdictions are required to ask participants the core instrument questions, and they can choose whether to include additional components.

### Caregiver status

The caregiver module in the BRFSS is an optional module that includes the following question to ascertain whether a participant is a caregiver: “During the past 30 days, did you provide regular care or assistance to a friend or family member who has a health problem or disability?”

Participants answered either yes or no, or the interviewer coded the response as “don’t know,” “care recipient died within 30 days,” or “refused to answer the question.” Participants who answered yes were classified as caregivers and those who said no or indicated that their care recipient died within the last 30 days were classified as noncaregivers. Those who answered “don’t know” or refused to answer were recoded as missing.

### Caregiver experience

Participants classified as caregivers were asked several questions pertaining to their role as a caregiver. The following items were included in the final analysis as a method for assessing the caregiver experience:

1. Relationship between caregiver and care recipient (16 response options)
2. Duration of providing care (response options: <30 days, 1 to <6 months, 6 months to <2 years, 2 to <5 years, or ≥5 years)
3. Average hours of care provided per week (response options: 0–8, 9–19, 20–39, and ≥40)
4. Main health problem of the care recipient (16 response options)

Among caregivers, the care recipient’s relationship to the caregiver was categorized as parent, spouse or partner, other family member, or nonfamily member. Dichotomous variables to indicate caregiving duration (<2 years, ≥2 years) and caregiving hours (<20 hours per week or ≥20 hours, reflecting at least part-time work) were created. These classifications were based on previous categories used in reports based on the BRFSS Caregiver Module and were made to ensure an adequate number of respondents in each group.

To include the variable pertaining to the care recipient’s main health problem, we created a single dichotomous variable (low risk or high risk) based on the recipient’s risk for developing severe flu complications (25). The low-risk category included care recipients whose main health problem was arthritis or rheumatism, dementia or other cognitive impairment disorders, developmental disabilities, mental illnesses, substance abuse or addiction disorders, and injuries. The high-risk category included care recipients whose main health problem was asthma, cancer, chronic respiratory conditions, diabetes, cardiovascular disease, HIV infection, old age/infirmity/frailty, other organ disease (eg, renal failure), and other unspecified medical conditions. The “other” response to this question was placed in its own category coded “unknown risk of developing severe flu complications.”

### Influenza vaccine

Influenza vaccine status is included on the core portion of the BRFSS and ascertained using the following question: “During the past 12 months, have you had either a flu shot or a flu vaccine that was sprayed in your nose?”

Participants answered either yes or no, or the interviewer coded the response as “don’t know” or “refused to answer.” Participants whose responses were coded as “don’t know” and those who refused to answer were recoded as missing data, and the variable was kept as dichotomous.

### Sociodemographic and health-related variables

On the basis of existing literature related to vaccine uptake and preventive health behaviors (8), we considered 2 categories of independent variables: sociodemographic and health-related. The sociodemographic variables included age, sex, marital status, household income, educational attainment, and race and ethnicity. Health-related variables included whether the participant had a doctor’s visit within the past 2 years, had health insurance coverage, and their self-reported health status (excellent, very good, good, fair, or poor). All variables were coded to be categorical, and most were recoded to have a binary response.

### Sample

A total of 28 jurisdictions (27 states and the District of Columbia) included the caregiving module at least once during the 3 study years. For states that asked the caregiving module in multiple years, data from the most recent year were included in the final analysis.

Analyses were conducted accounting for the complex survey design of the BRFSS. Each data set was appropriately weighted based on the year and survey version on which the caregiver module appeared. The appended data set included 173,945 records, of which 19,775 respondents were removed because of missing values. The final sample size included 154,170 respondents.

### Statistical analysis

Chi-square bivariate analyses were conducted to compare flu vaccine uptake, caregiver status, and selected sociodemographic and health status variables. Two binary logistic regression models were conducted to analyze the relationship between flu vaccine uptake and caregiver status. In the first model, we compared caregivers and noncaregivers, controlling for health-related factors such as recent doctor’s visits and self-reported health status and sociodemographic factors such as age, race and ethnicity, gender, and education.

In a second model, we included only caregivers to determine whether flu vaccine uptake rates were affected by caregiving characteristics such as hours of care provided per week and how many years care had been provided. We tested for interactions between caregiving and sex and between caregiving and race and ethnicity, given that there are known differences in flu vaccination among these groups and that evidence suggests that caregiving has differential effects based on sex and race and ethnicity. We included multiplicative terms and considered a *P* value < .05 to be significant. For each variable in the logistic regression models, odds ratios, 95% CIs, and *P* values were reported. For binary analyses, a post-hoc power analysis demonstrated 100% power to detect a difference of 5% in flu vaccination between caregivers and noncaregivers with an α of 0.01 and 90% power to detect a vaccination difference of 1%. All analyses were conducted using survey (svy) commands with subpopulation statements as appropriate in Stata version 15 (StataCorp LLC). This study was determined to be exempt by the New York Medical College institutional review board.

## Results

Of the 154,170 included respondents, 20.5% identified as caregivers. The highest proportion of respondents in this study population were aged 65 years or older (19%). The age group with the lowest proportion of respondents for the whole study population was 18 to 24 years, at 13%.

Most respondents indicated their race or ethnicity to be non-Hispanic White (59%). Approximately 20% of respondents indicated they were of Hispanic or Latino origin, and 11% identified as non-Hispanic Black. The racial or ethnic group with the lowest proportion of respondents was the other or multiracial group, at 4%. Most (55.6%) respondents were female, and more than half of the respondents (52.3%) reported they were married.

Compared with noncaregivers, caregivers were more likely to have fair or poor self-reported health status (*P* < *.*001), be female (*P* < *.*001), be married or partnered (*P* = *.*04), and have at least a high school diploma (*P* < *.*001) (Table 1).

**Table 1:** Bivariate Analysis of Sociodemographic Characteristics and Health-Related Variables, Study on Flu Vaccine Uptake Among Caregivers and Noncaregivers, Behavioral Risk Factor Surveillance System, 2016–2018

Variable	Caregiver, weighted %	Noncaregiver, weighted %	*P* value^a^
**Self-reported health status**			
Fair or poor	20.1	17.2	<.001
Good or excellent	79.9	82.8	
**Age, y**			
18–24	10.8	12.8	.03
25–34	14.8	17.7	<.001
35–44	14.3	17.0	<.001
45–54	20.5	16.0	<.001
55–64	20.0	16.0	<.001
≥65	19.6	20.4	.22
**Sex**			
Male	41.3	50.6	<.001
Female	58.7	49.4	
**Marital status**			
Married	57.4	55.4	.04
Not married	42.6	44.6	
**Household income, $**			
<50,000	51.3	49.9	.21
≥50,000	48.7	50.1	
**Education**			
Less than high school	10.6	14.6	<.001
High school or more	89.4	85.4	
**Race and ethnicity**			
Asian, non-Hispanic	2.6	6.9	<.001
Black, non-Hispanic	11.4	10.3	.07
Hispanic, any	16.2	20.3	<.001
Other, multiracial, non-Hispanic	4.6	3.2	<.001
White, non-Hispanic	65.1	59.3	<.001

a *P* value based on the χ^2^ test of weighted proportions.

For the whole study population and within the caregiver and noncaregiver groups, approximately 36% of respondents reported receiving a flu vaccine within the past 12 months. There was no difference in the prevalence of receiving a flu vaccine by caregiver status (*P* = *.*95). A higher proportion of female respondents (39.5%) than male respondents (32.7%) had received a flu vaccine within the last 12 months (*P* < .001).

On the basis of the first adjusted logistic regression model, caregivers and noncaregivers had similar odds of receiving a flu vaccine in the past year (adjusted odds ratio [AOR] = 0.98; 95% CI, 0.90–1.07; *P* = *.*62) (Table 2). Although caregiver status was not significantly associated with flu vaccine uptake, health-related variables and several sociodemographic factors were significantly related to flu vaccination (Table 2). Respondents who were aged 65 years or older, female, married, and living in households with an income of $50,000 or more had higher odds of receiving a flu vaccine within the past year (*P* < *.*001 for all). Respondents who identified as non-Hispanic Black had nearly 30% lower odds of receiving a flu vaccine than non-Hispanic White respondents (AOR = 0.70; 95% CI, 0.62–0.79; *P* < *.*001). There were no significant interactions between sex or race of the caregiver with flu vaccine uptake.

**Table 2:** Logistic Regression for Odds of Receiving a Flu Vaccine, Study on Flu Vaccine Uptake Among Caregivers and Noncaregivers, Behavioral Risk Factor Surveillance System, 2016–2018

Variable	Adjusted odds ratio (95% CI)	*P* value^a^
**Caregiver status**		
Caregiver	0.98 (0.90–1.07)	.62
Noncaregiver	1 [Reference]	—
**Self-reported health status**		
Fair or poor	1.21 (1.11–1.33)	<.001
Good or excellent	1 [Reference]	—
**Duration since last doctor’s visit**		
Within last year	2.08 (1.88–2.29)	<.001
More than 1 year ago	1 [Reference]	—
**Health insurance status**		
Has health insurance	1.73 (1.45–2.05)	<.001
Has no health insurance	1 [Reference]	—
**Age, y**		
≥65	2.36 (2.18–2.55)	<.001
<65	1 [Reference]	—
**Sex**		
Female	1.25 (1.16–1.35)	<.001
Male	1 [Reference]	—
**Marital status**		
Married	1.26 (1.17–1.36)	<.001
Not married	1 [Reference]	—
**Household income, $**		
≥50,000	1.25 (1.15–1.36)	<.001
<50,000	1 [Reference]	—
**Education**		
High school education or more	1.09 (0.94–1.26)	.26
Less than high school education	1 [Reference]	—
**Race and ethnicity**		
Asian, non-Hispanic	1.15 (0.90–1.47)	.25
Black, non-Hispanic	0.70 (0.62–0.79)	<.001
Hispanic, any	0.92 (0.81–1.04)	.18
Other, multi-racial, non-Hispanic	0.98 (0.84–1.14)	.79
White, non-Hispanic	1 [Reference]	—

a *P* value based on survey weighted logistic regression model.

In the second regression model, which only included caregivers, weekly hours of caregiving, duration of caregiving, and caregiving relationship had no significant effect on flu vaccine uptake. Additionally, no significant difference in flu vaccine uptake was found between caregivers who cared for people who had a higher risk of developing severe flu complications versus having a lower risk of developing severe flu complications (AOR = 1.08; 95% CI, 0.88–1.29; *P* = *.*50) (Table 3). Apart from sex, the AORs and *P* values for the health-related and sociodemographic variables in this model were similar to those in the model that included both caregivers and noncaregivers.

**Table 3:** Logistic Regression for Odds of Receiving Flu Vaccine Among Caregivers, Controlling for Caregiver Characteristics, Study on Flu Vaccine Uptake Among Caregivers and Noncaregivers, Behavioral Risk Factor Surveillance System, 2016–2018

Variable	Adjusted odds ratio (95% CI)	*P* value^a^
**Weekly hours providing care**		
≥20	1.02 (0.84–1.24)	.81
<20	1 [Reference]	—
**Duration of caregiving, y**		
≥2	1.11 (0.95–1.30)	.20
<2	1 [Reference]	—
**Care recipient’s risk for developing severe flu complications^b^**		
High	1.07 (0.88–1.29)	.50
Unknown	1.12 (0.91–1.37)	.29
Low	1 [Reference]	—
**Relationship to care recipient**		
Spouse or domestic partner	0.88 (0.68–1.14)	.33
Other relative	0.92 (0.74–1.15)	.48
Nonrelative	0.87 (0.68–1.10)	.25
Parent or parent-in-law	1 [Reference]	—
**Self-reported health status**		
Fair or poor	1.32 (1.09–1.58)	.004
Good or excellent	1 [Reference]	—
**Duration since last doctor’s visit**		
Within last year	1.98 (1.62–2.43)	<.001
More than 1 year ago	1 [Reference]	—
**Health insurance status**		
Has health insurance	1.70 (1.13–2.54)	.01
Has no health insurance	1 [Reference]	—
**Age, y**		
≥65	2.32 (1.90–2.83)	<.001
<65	1 [Reference]	—
**Sex**		
Female	1.09 (0.93–1.27)	.28
Male	1 [Reference]	—
**Marital status**		
Married	1.28 (1.07–1.52)	.005
Not married	1 [Reference]	—
**Household income, $**		
≥50,000	1.21 (1.02–1.43)	.03
<50,000	1 [Reference]	—
**Education**		
High school education or more	1.07 (0.78–1.46)	.68
Less than high school education	1 [Reference]	—
**Race and ethnicity**		
Asian, non-Hispanic	1.03 (0.54–1.99)	.93
Black, non-Hispanic	0.72 (0.56–0.94)	.02
Hispanic, any	0.83 (0.60–1.15)	.26
Other, multi-racial, non-Hispanic	0.86 (0.63–1.17)	.33
White, non-Hispanic	1 [Reference]	—

a *P* value based on survey weighted logistic regression model.

b Conditions considered low risk for severe flu complications include arthritis/rheumatism, dementia or other cognitive impairment disorders, developmental disabilities (such as autism, Down Syndrome, and spina bifida), mental illnesses (such as anxiety, depression, or schizophrenia), substance abuse or addiction disorders, and injuries. Conditions considered high risk for severe flu complications include asthma, cancer, chronic respiratory conditions (such as emphysema or chronic obstructive pulmonary disease), diabetes, heart disease, hypertension, stroke, HIV, other organ failure (such as kidney or liver failure), and old age/frailty. Other was considered its own category of unknown risk.

## Discussion

The primary goal of this study was to determine whether a difference existed in flu vaccine uptake between caregivers and noncaregivers. We hypothesized that caregivers would have a lower rate of flu vaccine uptake than noncaregivers. The rationale behind this hypothesis was derived from the literature that suggests that caregivers practice less self-care (26). However, our analysis indicated no significant difference in flu vaccine uptake between caregivers and noncaregivers. Flu vaccine uptake was low among all adults, with fewer than 4 in 10 caregivers and noncaregivers receiving it in the past year. This similarity between caregivers and noncaregivers could suggest that some barriers to vaccination among caregivers, whom we expected would face caregiving-related barriers to self-care, had been removed, enabling them to achieve a similar level of vaccine coverage as their peers. Characteristics of the caregiving experience (duration of care, relationship to care recipient, and hours of care provided weekly) were also not associated with flu vaccine uptake.

Several sociodemographic characteristics were associated with flu vaccine uptake in the study. Black respondents had the lowest odds of all racial groups of obtaining a flu vaccine compared with non-Hispanic White respondents. This finding is consistent with previous literature that suggests a disparity in vaccine uptake between Black or African American people and people of other races (17). Respondents aged 65 years or older had more than twice the odds of obtaining a flu vaccine compared with respondents younger than 65 years, consistent with past research (16).

Respondents who reported having health insurance had significantly greater odds of obtaining a flu vaccine than those without health insurance. This finding is plausible as the Patient Protection and Affordable Care Act covers preventable health services, including immunizations, under its 10 Essential Health Benefits (27). All health insurance plans must include coverage for immunizations, including the flu vaccine, but issues of health equity and access can contribute to the overall low uptake of the flu vaccine.

The final result in our analysis was about self-reported health status. Respondents who reported their health status to be fair or poor had 21% higher odds of receiving a flu vaccination within the past year. This finding is consistent with recent research that investigated the relationship between self-reported health status and flu vaccine uptake using BRFSS data (18).

Caregivers’ uptake of flu vaccination did not differ by the care recipients’ susceptibility to flu. We expected that caregivers would take extra precautions to protect their care recipients from developing any further illness. Perhaps caregivers are unaware of their care recipient’s risk for developing severe flu complications, do not perceive that they are at high risk of transmitting it, or do not have access to a flu vaccine. Alternatively, the care recipient may have received the flu vaccine and the caregiver does not see the value in being vaccinated. To our knowledge, however, no study has yet investigated caregivers’ perceptions about their risk or their care recipients’ risks of contracting flu or experiencing severe illness because of flu infection.

The finding that there is no difference between flu vaccination uptake between caregivers and noncaregivers is of some concern. Given that caregivers interact with people who need assistance, often because of health conditions, having a vaccinated caregiver population could protect the most vulnerable members of the population against a common infectious disease. Also, flu infection in caregivers could render them incapable of providing care while they recover, which might have negative consequences for care recipients. Therefore, flu vaccine uptake should be higher in the caregiver population.

Although our analysis did not find any significant difference in flu vaccine uptake between caregivers and noncaregivers, there are policy and practice implications to consider. Given the concerningly low rates of flu vaccine coverage in the caregiver and noncaregiver populations, many opportunities in practice settings can improve this. One method would be to provide incentives to people who receive the annual flu vaccine. The use of mobile technology reminders and incentives could extend the reach of public and community health advocates to improve flu vaccine uptake (28).

Since many people in the US are covered by private (nongovernment) insurance policies, providing incentives may be possible through reductions in premium, copayment assistance, or other health care–specific incentives, such as discounts for wellness products (29). Another potential remedy could be offering the flu vaccine to a patient’s caregiver during outpatient visits at no or modest cost. The care recipient’s insurance provider may cover this low-cost vaccine for the caregiver to prevent a flu-related doctor’s or hospital visit for the policy holder. The logistics of implementing a similar program with adults and their caregivers would be more complicated and would require buy-in from health insurance agencies.

The results and insights of this study add to the ever-growing body of caregiver research and provide direction for future research and policy initiatives to improve the health and well-being of caregivers and their recipients. Because the final data set included records from several appended BRFSS data sets, the sample size for the study was large and representative of the US population. Twenty-seven states and the District of Columbia were included in the final analysis, which promoted heterogeneity and generalizability of the results.

However, our study has limitations. The data used were secondary data based on self-reported multiple-choice responses, and the study characteristics were limited to the data collected in the survey. Other than asking whether a respondent obtained a flu vaccine within the last year, no additional questions regarding the flu vaccine were asked in the BRFSS. Medical records were not used to confirm receipt of a flu vaccine, which meant that survey administrators had to take respondents at their word. Because of the cross-sectional nature of the study, causality cannot be inferred from the results. We also did not find any relationship between caregiver status and flu vaccine uptake. 

Respondents who disclosed that they provide care were only able to report the main condition their care recipient had and not any additional comorbidities that may place their charge at risk for developing severe flu complications. This may have resulted in the caregiver being misclassified as assisting someone with “low risk for developing severe flu complications.” Misclassification or bias also may have resulted from the self-reported nature of BRFSS data and the potential for recall bias around the receipt of the flu vaccine. Additionally, we acknowledge that the variables used in the study regarding caregiver characteristics such as duration of caregiving and weekly hours of providing care do not accurately reflect caregiver burden as described in the literature (6). The BRFSS does not include a measure of caregiver burden. Therefore, we were unable to measure this construct directly in this study and to assess whether it affected caregivers’ receipt of the flu vaccine. To control for the variabilities in caregiving, our inclusion of these variables was to demonstrate how different caregiver experiences may influence vaccine uptake.

Finally, some respondents who indicated that they did not provide regular care or assistance to a friend or family member may not have recognized their potential role as a caregiver. For example, a respondent who completes some household tasks for a family member may not recognize those as providing care and assistance. This factor may have resulted in caregivers being misclassified as noncaregivers, which could attenuate any true relationship.

More research is needed to understand why flu vaccine uptake is low in the caregiver population. Understanding perceived barriers caregivers have about receiving the flu vaccine through qualitative studies would provide insights into how to address vaccine hesitancy, dispel misconceptions, improve the flu vaccine uptake, and decrease the overall impact the flu has in the population. Longitudinal studies could help determine whether taking on the caregiver role changes the likelihood that an individual receives the flu vaccine or how caregiver burden affects vaccine uptake. Additional research investigating the impact of existing state flu vaccine policies and laws can yield insights on caregiver vaccine uptake behaviors.

Our study provides a valuable insight into pre–COVID-19 vaccination practices, which may guide future vaccination practices, recommendations, and policies. Future research initiatives can include investigating the uptake of other vaccines in caregivers. Examples could include the hepatitis A, hepatitis B, shingles, and pneumococcal vaccines. Investigating the uptake of the SARS-CoV-2 vaccine in caregivers (family and professional) in future studies is important. Although the data analyzed in this study predated the COVID-19 pandemic, no evidence suggests that caregivers have been more resistant than noncaregivers to be vaccinated. The disparity in flu vaccine uptake found in this study is consistent with what is known about vaccine hesitancy, including questioned effectiveness, safety, and necessity (17). Future research might explore the impact of the political environment on vaccine uptake (30).

Our study indicates that gaps exist in our understanding of caregiver primary prevention strategies, specifically regarding flu vaccinations. We noted no difference in the flu vaccine uptake rates in caregivers and noncaregivers. Given the role caregivers fulfill in the health care system, flu vaccine uptake should ideally be higher in the caregiver population than the general population. Further outreach and intervention are needed to improve flu vaccine rates in caregivers.
